# Quantitative Assessment of Autonomic Regulation of the Cardiac System

**DOI:** 10.1155/2019/4501502

**Published:** 2019-04-21

**Authors:** Jian Kang Wu, Zhipei Huang, Zhiqiang Zhang, Wendong Xiao, Hong Jiang

**Affiliations:** ^1^The University of Chinese Academy of Sciences, Beijing, China; ^2^Institute of Healthcare Technologies, Chinese Academy of Sciences, Nanjing, China; ^3^University of Leeds, West Yorkshire, UK; ^4^Beijing University of Science and Technology, Beijing, China; ^5^China-Japan Friendship Hospital, Beijing, China

## Abstract

Autonomic neural system (ANS) regulates the circulation to provide optimal perfusion of every organ in accordance with its metabolic needs, and the quantitative assessment of autonomic regulation is crucial for personalized medicine in cardiovascular diseases. In this paper, we propose the Dystatis to quantitatively evaluate autonomic regulation of the human cardiac system, based on homeostatis and probabilistic graphic model, where homeostatis explains ANS regulation while the probability graphic model systematically defines the regulation process for quantitative assessment. The indices and measurement methods for three well-designed scenarios are also illustrated to evaluate the proposed Dystatis: (1) heart rate variability (HRV), blood pressure variability (BPV), and respiration synchronization (Synch) in resting situation; (2) chronotropic competence indices (CCI) in graded exercise testing; and (3) baroreflex sensitivity (BRS), sympathetic nerve activity (SNA), and parasympathetic nerve activity (PNA) in orthostatic testing. The previous clinical results have shown that the proposed method and indices for autonomic cardiac system regulation have great potential in prediction, diagnosis, and rehabilitation of cardiovascular diseases, hypertension, and diabetes.

## 1. Introduction

Autonomic neural system (ANS) regulates the circulation to provide optimal perfusion of every organ in accordance with its metabolic needs. Together with the endocrine and immunological systems, it adjusts the internal environment of the organism to respond the changes in the external environment [1]. Therefore, understanding the ANS and the way it regulates body circulation is crucial for personalized medicine in cardiovascular diseases. The understanding of the ANS regulation in the cardiac system can be traced back to the findings of two Nobel Prize winners: (1) Corneille Heymans in 1938 identified the carotid sinus nerves [2], which are tiny baroreceptor and chemoreceptor nerves and can sense changes in hemodynamic pressure and humoral factors and send output to the sympathetic and parasympathetic nerves, and (2) Axelrod [3], Von Euler [4], and Del and Katz [5] identified acetylcholine (ACh) as a transmitter for the parasympathetic nerves, norepinephrine (NE), and sympathetic nerves. However, the ANS regulation of the cardiac system can be viewed as a complex dynamic system, and it can be well described by “Homeostasis” [6], which is now regarded as one of the core competencies by the American Association of Medical Colleges and Howard Hughes Medical Institute and a core concept necessary for future physicians [7].

In clinical settings, autonomic dysfunction has been linked to direct detrimental effects towards heart failure and chronic kidney disease [8]; thus, quantitative methods to evaluate the ANS regulation has great potential to generate innovative diagnostic and treatment approaches that limit hypertension and target end-organ damage. Recent research has shown that the autonomic neurohumoral system can dramatically influence morbidity and mortality from cardiovascular disease through influences on the innate and adaptive immune systems [9]. Due to the high metabolic rate of brain tissue, the precise regulation of cerebral blood flow (CBF) is critical for maintenance of constant nutrient and oxygen supply to the brain [10]. The metabolic syndrome is characterized by the clustering of various common metabolic abnormalities in an individual, which is also associated with increased risk for the development of type 2 diabetes and cardiovascular diseases. The augmented sympathetic activity in individuals with metabolic syndrome worsens prognosis of this high-risk population [11]. Experimental and clinical investigations have validated the hypothesis: the origin, progression, and outcome of human hypertension are related to dysfunctional autonomic cardiovascular control, which is particularly true for abnormal activation of the sympathetic division [12].

Since the quantitative assessment of the autonomic regulation is tremendously important for clinical and healthcare applications, there is urgent need to quantitatively evaluate the ANS regulation status. Unfortunately, there are only two invasive methods to measure certain aspects of the ANS thus far: (1) microneurography to assess muscle sympathetic nerve activity and (2) the norepinephrine isotope dilution to determine noradrenalin in the blood to evaluate spillover of the sympathetic nervous system [13]. Although HRV is an indirect biomarker of the cardiac autonomic nervous system activity [14], ANS regulation of the cardiac system is complex in nature and existing HRV assessment is rather ad hoc without any theoretical model. Therefore, HRV indices obtained in different settings and by different persons are often inconsistent, resulting in difficulties for clinical interpretation.

In summary, ANS regulation of the cardiac system plays a central role in both research and clinical practices, and we will focus on the quantitative assessment of autonomic regulation of the cardiac system in this paper. The main contributions are as follows:

- We propose the Dystatis to quantitatively evaluate autonomic regulation of the human cardiac system, based on homeostatis and the probabilistic graphic model, where homeostatis explains ANS regulation while the probability graphic model systematically defines the regulation process for quantitative assessment.
- The Dystatis is elaborated in three well-designed scenarios, where indices and measurement methods for each scenario are also proposed and illustrated by clinical applications:
  1. HRV, BRV, and Synch in resting situation: Dystatis provides theoretical model and guidelines for the test design and data processing and interpretations, in order to solve existing inconsistence problems.
  2. CCI in graded exercise testing: Dystatis metabolic requirement is enlarged by graded exercise so that CCI can be obtained without considering effects from other internal and external interactions. These are minor compared to graded exercises.
  3. BRS, SNA, and PNA in orthostatic testing: based on Dystatis, orthostatic testing creates a large blood pressure drop and then a large BRS output to sympathetic and parasympathetic nerves. As such, the mathematical model for solution of BRS, SNA, and PNA can be greatly simplified by neglecting other internal and external interactions in the ANS regulation.

## 2. Dystasis: Systematic Quantitative Assessment Methodology for ANS Regulation of the Cardiac System

Human body is a complex biological system, of which homeostasis is a crucial property in maintaining the life. It is the self-regulating process by which biological systems maintain stability in order to adjust to conditions that are optimal for survival. The stability attained is a dynamic equilibrium, in which continuous change occurs yet relatively uniform conditions prevail.

Dystasis is built up on homeostasis and defined as follows: ANS regulation of the cardiac system is a part of body's complex biological system. Through ANS self-regulating process, the cardiac system tends to reach and maintain a dynamic equilibrium state, in order to supply cells and organs with their metabolic needs, e.g., oxygen, nutrients, and removal of waste, survive in various internal and external environments, and support various physical and mental activities. The characteristics of Dystasis are (1) equilibrium: the ANS self-regulating process of the cardiac system reaches and maintains an “equilibrium” state in a relative steady internal and external environment, with no or minor changes in terms of physical and mental activities. The property and its numerical measures of the state of this equilibrium of the individual's ANS self-regulating process shall provide quantitative performance evaluation of how well one's ANS regulation system works; (2) dynamic: the ANS self-regulating process of cardiac system should be “dynamic” enough, being able to work in dynamic environment, support various physical and mental activities of the body, and defend virus invasions. In other words, it should be able to reach new equilibrium state as soon as possible when there is a change of internal/external environment or physical/mental activities. For instance, ANS regulation interacts with the immune system to control inflammation [15] and ANS regulation of the cardiac system increases oxygen supply and reaches a new equilibrium when the intensity of physical activity increases to a new level. The capability of ANS regulation to accommodate changes of internal and external environment, as well as activity needs, is another important measure.

**Figure 1:**
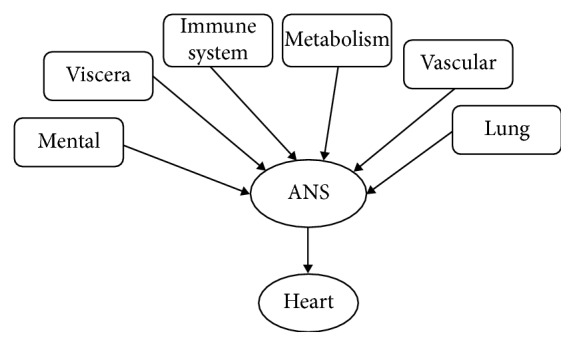
Probabilistic graphic model of autonomic regulation of the cardiac system with internal and external influences.

In order to quantitatively evaluate the state and capability of ANS regulation of the cardiac system, one feasible approach is the probabilistic graphic model-based approach [16], as shown in Figure 1. Principally, the interactions of ANS with cardiac, respiration, vascular, metabolic, immune, viscera, and mental systems are bidirectional [17]. Here, in Figure 1, the objective is to estimate ANS state through all possible observations, in case of ANS regulation of the heart rate with major internal and external influences; i.e., RSA is a terminology for heart rate modulation by respiration; blood pressure formed in the vascular system and sensed by baroreflex which then affects the heartbeat; physical activities stimulate metabolic needs and increase the heart rate; inflammation in the immune system breaks the stability of ANS regulation and then heart rate variations; the dorsal vagal complex is responsible for the interaction between viscera organs and ANS; and the ventral vagal complex is responsible for mental activities [17]. The observation of ANS regulation here is variations of the heart rate, blood pressure, and respiration. The sympathetic innervation of the heart and blood vessels is excitatory. It stimulates vasoconstriction and increases the heart rate and cardiac contraction. On contrary, the parasympathetic vagal innervation is inhibitory, which decreases the heart rate and cardiac contraction. The balance of the two appears as variations of the heart rate and blood pressure and can be characterized by indices which represent properties and rules of those variations caused by regulation: The sympathetic activity increases during the flight-or-fight response, whereas parasympathetic activity increases to calm the heart when there appears emotionally driven high blood pressure.

For the estimation purpose and from Figure 1, we can obtain the following formula, via probabilistic graphic model:

(1) $$\begin{array}{c} P\;=\;p\left(\text{heart}/A\right)\;\;p\left(A/\text{mental}\right)\;\;p\left(A/\text{viscera}\right)\;\;p\left(A/\text{immune}\right)\;\;p\left(A/\text{metabolic}\right)\;\;p\left(A/\text{vascular}\right)\;\;p\left(A/\text{lung}\right), \end{array}$$

where *A* is the state of ANS to be estimated through observations connected with ANS in the graph of Figure 1. However, not all nodes connected with the ANS node are observable or measurable. For the quantitative assessment purpose, it is the best to intentionally create assessment scenario where the influences of the measurable nodes are maximized whilst minimizing those of the unmeasurable nodes. Therefore, we designed the following three assessment scenarios:

1. Variability of the heart rate and blood pressure (HRV and BPV) while the subject is in resting or other steady state: the ideal measurement scenario is zero or known steady physical activity, minimal mental activity, and minimal viscera disturbance. The variability indices are used to characterize the state of equilibrium of individual's ANS self-regulating process, which directly reflects states of immune system, linking with inflammation biomarkers.
2. CCI in graded exercise testing: the effect of physical activity on ANS is maximized so that the influences from the rest sources can be neglected. CCI provide numerical measures to characterize the capability of ANS regulation to accommodate changes of exercise intensity.
3. BRS, SNA, and PNA are obtained by model-based analysis of blood pressure (BP) and heart rate (HR) pairs acquired in orthostatic testing: Via orthostatic test, large blood pressure drops around 30 mmh is obtained. The input from baroreflex to SNA and PNA becomes the major effect, and the rest can be neglected. As such, the mathematical model for the solution can be simplified as a subgraph of the graphic model in Figure 1.

## 3. Variabilities in Resting or Steady Testing Scenario

The indices of HRV and BPV consist of time-domain second-order statistics, for example, standard deviation of ECG normal-to-normal intervals (SDNN) and standard deviation of differences of neighboring normal-to-normal intervals (SDSD). Frequency-domain indices are calculated at very low frequency band (VLF, 0.004–0.04 Hz), low frequency band (LF, 0.04–0.15 Hz), and high frequency band (0.15–0.4 Hz). The problem is then to quantitatively evaluate the state of ANS and infer the physiological and psychological implications, given measured variabilities of the heart rate, blood pressure, respiration, and assessment scenario that the physical activity is zero or constant. Based on Dystasis framework, according to equation (1) and graphic model in Figure 1, there are still three nodes: mental activities and the states of viscera organs are not known or unmeasurable and inflammations in the immune system are the ones to be inferred. Now, in this assessment scenario, in order to obtain the stable and consistent quantitative measures, we have to minimize the influences of mental activities and viscera organs. To fulfill this requirement, variabilities are best to be measured when the subject is in deep sleep or in a coherence state between respiration and heart rate where mental activities and viscera influences are purposely minimized.

HRV has been studied for a long time to reflect the states of ANS regulation [14, 18]. In clinical practice, HRV is usually evaluated using Holter device and software, without consideration of physical activities and other influences. This has resulted in inconsistences in various studies and limited the clinical applications of HRV. To quantitatively evaluate the physical activities and define the testing scenario, in case of using Holter device, a three-dimensional accelerometer sensor is used to detect and classify posture and activity into laying, sitting or standing, walking, or running. HRV indices are then calculated when any of those postures and activities keeps for more than 10 minutes [19].

The interaction between heartbeat and respiration is the well-known respiratory sinus arrhythmia (RSA). The wisdom of the body to maintain the homeostasis is achieved by synchronizing heartbeats with breathing and consequently to maximize the efficiency of the cardiopulmonary system in metabolic and circulation process. This equilibrium state is the result of resonance of the cardiopulmonary system. There are indices proposed to evaluate the degree of the resonance of the cardiopulmonary system. The most common used one is coherence measure (Coh), the cross power spectral density of the heart rate and respiration signals [20].

The resonance of the cardiopulmonary system represents the equilibrium of ANS regulation, where one reaches both physiological and psychological healthy state. Therefore, Coh can be used to compose numerical measures to visually represent one's health state, especially psychological health state, and then, variability-biofeedback training is used to help one to gain resonance state. A clinic trial was conducted in the University of Chinese Academy of Sciences (UCAS) Hospital to test the effectiveness of HRV biofeedback (HRVB) for pregnant women in managing anxiety and depression [21]. 20 pregnant women at last trimester (28–32^th^ week) without pregnancy-induced hypertension and diabetes were randomly assigned to the HRVB group and the control group. Participants in the HRVB group practiced HRVB for 30 minutes per day, while participants in the control group did not. Following checks are conducted for all participants every two weeks: blood pressure (BP), fasting blood glucose (FBG), HRV of pregnant women (PHRV) and their fetuses (FRHV), and subjective assessment on pressure using Pregnancy Pressure Scale (PPS), depression using Edinburgh Postnatal Depression Scale (EPDS), and sleep quality using Pittsburgh Sleep Quality Index (PSQI). The clinical trial continued for subjects until they are in hospital for delivery. In the trial, the HRVB group has shown significant improvement over the control group with respect to blood pressure stability (*p* > 0.05), depression reduction (*p*=0.013), and sleep quality improvement, while fetuses in the HRVB group has shown significant improvement with respect to HRV SDNN (*p* < 0.01) and LF spectrum power (*p* < 0.01).

HRV and BPV can be used as a noninvasive assessment tool for autonomic nervous system function, and reduced and/or abnormal HRV and BPV are associated with increased risk of mortality in cardiac patients. For both adults and children, increased blood pressure variability (BPV) appears to be directly related to sympathetic overactivity with increased risk of end-organ damage and cardiovascular events. Decreased HRV has been observed in adults and children with chronic kidney disease and is an independent predictor of mortality [22].

Autonomic dysfunctions are the most common nonmotor symptoms of Parkinson's disease (PD) and often precede the motor symptoms of the disease. Clinical study has shown that HRV and BPV can be used as markers to indicate the treatment progress and stages of the disease [23].

A review of research literature [24] tells that affected central nervous system structures and implicated autonomic nervous system regulation coexist in Alzheimer's disease. Assessment of autonomic dysfunction can be used as an early marker of Alzheimer's disease and used for differential diagnosis among dementia subtypes.

## 4. Chronotropic Competence Indices in Graded Exercise Testing Scenario

Graded exercise tests, such as cardiopulmonary exercise test (CPX), have been used in clinical practice to test the exercise capability in terms of maximum oxygen metabolism [25]. In Dystasis family, CCI are designed to evaluate the capability of the ANS regulation of the cardiac system in response to exercise, where the subject does not necessarily reach the maximum exercise intensity.

Chronotropic incompetence (CI) is a terminology describing the status of attenuated heart rate response to exercises. CI has been studied for the last 50 years [26]. Typical CI-related measurements include the maximum heart rate and heart rate recovery after exercise. There have been a lot of research efforts to explore the usefulness of CI parameters in clinical applications, i.e., their diagnosis value of coronary artery [27], prognosis and management of heart failure [28, 29], diabetes [30, 31], and hypertension [32, 33]. Although CI is an independent predictor of major adverse cardiovascular events and overall mortality, the importance of CI is underestimated [34]; this may be in part due to multiple definitions, the confounding effects of aging and medications, and the need for formal exercise testing for definitive diagnosis.

We have formally defined CCI as part of Dystasis in a systematic way and in terms of ANS regulation capabilities and endowed CCI with clear physiological and clinical implications. CCI are defined as follows:

(1) Resting heart rate (HRrest) and resting blood pressure (BPrest): The resting heart rate and resting blood pressure are defined as the heart rate and blood pressure when a person is awake, in a neutrally temperate environment, and has not been subject to any recent exertion or stimulation, such as stress or surprise.
(2) Chronotropic rate (CR_HR_ and CR_BP_): chronotropic rate represents the rate at which the heart rate and blood pressure increase as exercise intensity increases. It is measured as the amount of heart rate or blood pressure increase in response to every unit of metabolic equivalent (MET) exercise intensity increase. In practice, it can be measured and calculated as
  (2) $$\begin{array}{c} {\text{CR}}_{\text{HR}}=\frac{\left({\text{HR}}_{\text{stage}}-{\text{HR}}_{\text{rest}}\right)}{\left({\text{MET}}_{\text{stage}}-1\right)},\\ {\text{CR}}_{\text{BP}}=\frac{\left({\text{BP}}_{\text{stage}}-{\text{BP}}_{\text{rest}}\right)}{\left({\text{MET}}_{\text{stage}}-1\right)}. \end{array}$$
CR_HR_ is similar with the “Exercise HR” in EACPR/AHA Joint Scientific Statement [25]. It directly relates to sympathetic nerves activation and provides insight into chronotropic competence and cardiac response to exercise. It normally increases ∼10 beats per MET. The chronotropic rate is an important parameter to provide personalized quantitative relation between HR and exercise intensity so that the target heart rate (THR) can be used to prescribe exercise intensity in exercise training. However, the chronotropic rate of a person may vary due to medication or rehab progress; it is recommended to measure the chronotropic rate promptly or monitor chronotropic rate changes in order to keep exercise prescription updated [35].
(3) Chronotropic limit (CL): chronotropic limit represents the maximal heart rate an individual can achieve without severe problems through exercise stress, as well as the blood pressure measured at the same time. It is measured as heart rate reserve and calculated as
  (3) $$\begin{array}{c} \text{CL}=\text{HRR}=\frac{\left({\text{HR}}_{\text{max}}-{\text{HR}}_{\text{rest}}\right)}{\left({\text{HR}}_{\text{PredM}}-{\text{HR}}_{\text{rest}}\right)}, \end{array}$$
  where HR_max_ is the maximal heart rate one achieves during the exercise test and HR_PredM_ is the predicted maximal heart rate, usually calculated as 220 − *a*ge. The maximal heart rate is usually obtained when reaching peak exercise, which can be identified during CPX testing. In this case, the normal value of CL is 0.8–1.3. However, when CPX testing or peak exercise is not achievable, then CL normal values are different for types of exercises. For example, in a 6-minute walking test, CL = 0.4 for a 60-year-old person should be considered normal. With a resting heart rate of 75 bpm, CR would be 10 beats per MET and the maximal heart rate would be 109 bpm with an exercise intensity of 4.4 MET.
(4) Chronotropic acceleration (CA): ANS requires certain time to adjust the heart rate and blood pressure to reach a new stable state or equilibrium when the exercise intensity increases to a new level in the graded exercise test. CA is defined as the time taken to reach new equilibrium after exercise intensity increases. CA is measured in seconds and represents the ability of the ANS regulation of the cardiac system in fulfilling metabolic needs.
(5) Chronotropic recovery at 1 minute after exercise (HR_recovery1_ and BP_recovery1_): it is defined as the reduction in the heart rate and blood pressure 1 minute after stopping exercise. The measurement of HR_recovery1_ and BP_recovery1_ requires the testee to try his best in the exercise, but not necessarily to reach one's maximum capacity. EACPR/AHA Joint Scientific Statement [25] considers that HR_recovery1_ provides insight into speed of parasympathetic reactivation and that the normal value of HR_recovery1_ should be > 12 beats. There have been a number of clinical studies on prognosis value of HR_recovery1_. For example, Dhoble et al. [36] examined conventional cardiovascular risk factors and exercise test parameters in 6546 individuals (mean age 49 years, 58% men) between 1993 and 2003. A total of 285 patients died during the follow-up period. HR_recovery1_ < 12 beats were found independently associated with mortality (*P* < 0.001).

A clinical trial in cardiac rehabilitation was conducted in Jiangsu Provincial Hospital to evaluate the usability of CCI [37], which are measured by Cardiac Chronotropic Competence Testing (3CT), a device produced by SmartHealth Electronics Ltd. 61 participants were recruited, including patients of unilateral ischemic or hemorrhagic stroke within the previous 6 months with some voluntary movement and preserved cognitive function. Participates are randomly assigned to the rehab group (30) and control group (31). Each patient from both groups was evaluated at the beginning and after 3 months using both subjective/qualitative and objective/quantitative measures, namely, the International Classification of Functioning, Disability and Health (ICF), and chronotropic competence indices (CCI) and 6 minute walking test (6MWT). Patients in the control group were given personalized rehab advices after the baseline test. Patients in the rehab group were equipped with a Microsens rehab assistant for regular rehab exercise at home. Personalized exercise prescription based on CCI is downloaded into MicroSens rehab assistant, which consists of rehab app on a smartphone and a wearable device.

Comparison between control and rehab groups after 3 months of rehab training using the *t*-test shows that, through out the rehab training, all the four ICF measurements, namely, walking, doing house-hold work, interpersonal interactions, and muscle power, have significant improvement (*p*=0.0070, 0.0209, 0.0089, and 0.0000, respectively). Consistently, after 3 months of rehab training, the rehab group is significantly better over the control group with respect to all three 3CT objective measures: 6-minute walking distance, chronotropic rate, and 1-minute heart rate recovery (*p*=0.0445, 0.0121, and 0.0414, respectively).

## 5. BRS, SNA, and PNA in Orthostatic Testing Scenario

Estimation of BRS, SNA, and PNA is carried out in orthostatic testing scenario where the subject is requested to suddenly stand up from a sitting position. As a result, blood pools in the vessels of the legs for a longer period and less is returned to the heart, thereby leading to a reduced cardiac output and fall in blood pressure. In order to counteract these changes, the frequency of afferent impulses in the aortic and carotid sinus nerves is reduced, which leads to parasympathetic withdrawal and sympathetic activation. Here, the nerve activity will be referred to as the baroreflex firing rate or simply the firing rate. Sympathetic activation leads to a growing release of norepinephrine which contributes to restoration of BP by increasing HR, cardiac contractility, and vasoconstrictor tone. In addition, parasympathetic withdrawal leads to decreased release of acetylcholine which also causes the increase of HR. This whole ANS regulation process can be described by a mathematical model [29, 38].

In the measurement, the subject wears a device which measures ECG, radial artery pulse wave and branchial artery pulse wave, and acceleration data to locate phases of the orthostatic posture. The orthostatic testing protocol is as follows:

1. The subject wears the device and sits on a chair, with the upper body straight up until reaching a stable state of heart rate
2. The subject stands up and keeps standing for 40 seconds
3. The above process is repeated for three times

**Figure 2:**
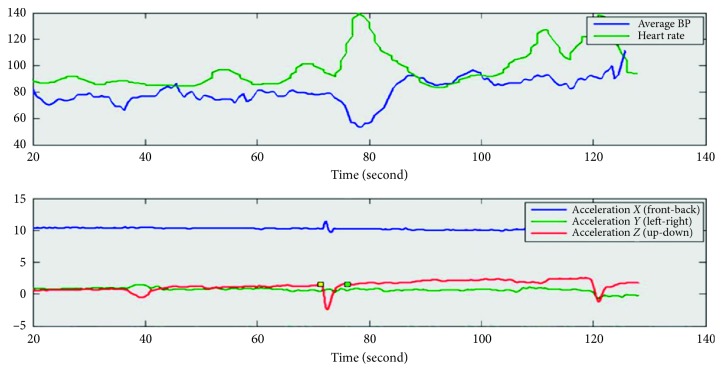
Curves of measured acceleration data (low), average blood pressure (up, blue), and heart rate (up, green). The stands up at the 47th second, when acceleration *Z* component (red) has a sudden drop, followed by average blood pressure fall and recovery and heart rate increase and recovery.

The device records all the data and sends the data to the computer wirelessly. The heart rate is calculated from ECG signal. The average blood pressure is estimated via pulse transmission time from radial artery pulse wave and branchial artery pulse wave with assumption that the physical properties of the blood vessel and the blood do not change within the measurement time.  Figure 2 shows a sample of the measurement data.

The blood pressure change in the orthostatic test is maximized, and the mathematical model defining the ANS regulation of heart rate due to blood pressure changes can then be simplified as a small subgraph of the probability graphic model in Figure 1. Based on the mathematical model, using a series of blood pressure and heart rate data pairs obtained in the orthostatic testing, we can perform the following.

**Figure 3:**
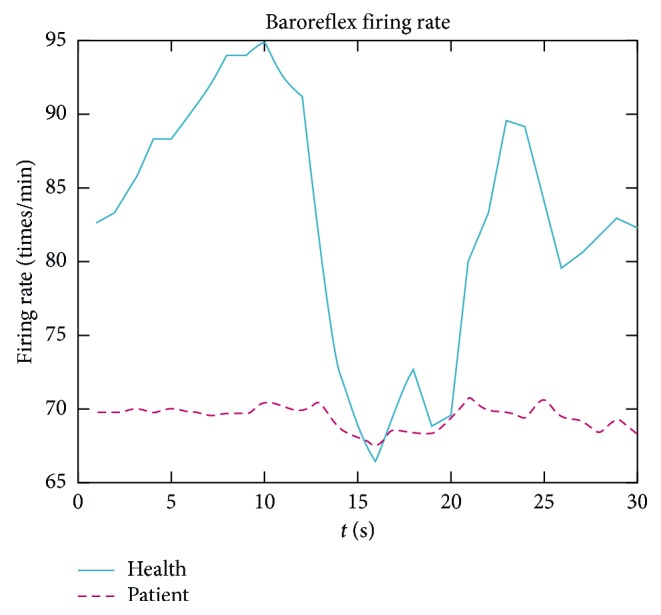
Baroreflex firing rate as a function of time. Results are shown from a healthy person (solid) and a hypertensive patient (dash).

- For each BP and HR pair, the following is performed:
  - BP is used to calculate the baroreflex firing rate
  - With baroreflex firing rate, sympathetic and parasympathetic outflows are predicted
  - Concentrations of noradrenaline and acetylcholine are computed as functions of the sympathetic and parasympathetic outflows
  - Heart rate is computed as a function of these two chemical concentrations
  - Computed HR is compared with the measured HR
- For all BP and HR pairs, optimization for the minimizing error is performed between computed HR and measured HR to get curves of baroreflex firing rate and sympathetic and parasympathetic outflows.  Figure 3 shows these curves for a healthy young person and a 50^th^ hypertension person. Other parameters, such as baroreflex sensitivity, can be derived from those curves and BP and HR data.

Noninvasive measurement of BRS, SNA, and PNA provides useful meanings to discover mechanisms that act to keep cerebral blood flow (CBF) constant, to understand immune system, for better management of metabolic syndrome and hypertension. The quantitative estimation of baroreflex sensitivity has been regarded as a synthetic index of neural regulation at the sinus atrial node, which has been shown to provide clinical and prognostic information in a variety of cardiovascular diseases, including myocardial infarction and heart failure [39]. Chronic hyperglycemia is the primary risk factor for the development of complications in diabetes mellitus (DM). Postprandial spikes in blood glucose, as well as hypoglycemic events, are blamed for increased cardiovascular events in DM. Glycemic variability (GV) includes both of these events. However, defining GV remains a challenge primarily due to the difficulty of measuring it [40]. A multicenter, prospective, open-label clinical trial including a total of 102 patients with type 2 diabetes [41] has found that GV was inversely related to BRS independent of blood glucose levels in type 2 diabetic patients and that measurement of BRS may have the potential to predict CV events in consideration of GV.

## 6. Conclusion and Remarks

We have described a systematic method for the quantitative assessment of autonomic cardiac system regulation, named Dystatis. The fundamental part of Dystatis is a quantitative assessment methodology based on homeostatis and the probabilistic graphic model, where homeostatis explains ANS regulation while the probability graphic model formally defines the regulation process and provides quantitative assessment basis. As instances of Dystatis, indices and measurement methods for three well-designed scenarios are also described together with clinical applications: (1) HRV, BPV, and Synch in resting situation, (2) CCI in graded exercise testing, and (3) BRS, SNA, and PNA in orthostatic testing.

Numerous clinical research results have shown that the proposed method and indices for autonomic cardiac system regulation have great application potential in the prediction, prognosis, and rehabilitation of cardiovascular diseases, hypertension, diabetes, and other autonomic nerves-related areas. Further researches are being carried out to work with various research institutions and hospitals to conduct multicenter clinical research to investigate potential applications of the proposed methods in the prediction, prognosis, and rehabilitation of cardiovascular diseases, hypertension, diabetes, and other autonomic nerves-related problems.

## Data Availability

The data used to support the findings of this study are available from the corresponding author upon request.
